# Model based noise correction enhances the accuracy of pancreatic CT perfusion blood flow measurements

**DOI:** 10.1038/s41598-025-24482-x

**Published:** 2025-10-23

**Authors:** Neha Vats, Philipp Mayer, Franziska Kortes, Miriam Klauß, Lars Grenacher, Hans-Ulrich Kauczor, Wolfram Stiller, Stephan Skornitzke

**Affiliations:** 1 Clinic for Diagnostic and Interventional Radiology (DIR), Heidelberg University Hospital, Heidelberg, Germany; 2 Department of Neuroimaging, Central Institute of Mental Health, Medical Faculty Mannheim, , Heidelberg University, Mannheim, Germany; 3 Radiology Rhein-Neckar, Schwetzingen, Germany; 4 MVZ Radiological and Nuclear Medicine Diagnostic Center, Conradia Radiology Munich, Munich, Germany; 5 Philips Healthcare, Hamburg, Germany

**Keywords:** Computed tomography perfusion, Image noise, Blood flow, Digital perfusion phantoms, Pancreas, Computed tomography, Pancreatic cancer

## Abstract

A model based noise correction algorithm was developed to improve the accuracy of CT perfusion (CTp) blood flow (BF) measurements affected by image noise. The algorithm used tissue attenuation curves (TACs), generated by convolving an impulse response function (IRF) with an arterial input function (AIF) averaged from 59 patient datasets. Gaussian noise was introduced to simulate noise, and BF was measured using deconvolution. The algorithm iteratively compared BF without added noise against noise-impacted BF to estimate ground-truth BF (GTBF). Performance was evaluated with digital perfusion phantoms (DPPs) for GTBF values of 5–420 ml/100 ml/min and added noise (standard deviation 25 HU), measuring absolute difference from GTBF and contrast-to-noise ratio (CNR). For clinical evaluation, CTp data from 14 pancreatic ductal adenocarcinoma (PDAC) patients was used. For DPPs, noise-impacted and noise-corrected BF were 140 ± 111 ml/100 ml/min and 131 ± 125 ml/100 ml/min, compared to GTBF of 131 ± 127 ml/100 ml/min. Post-correction, the absolute difference reduced from 18.8 to 3.6 ml/100 ml/min, with CNR improving from 2.52 to 2.66. In clinical datasets, BF for parenchyma shifted from 148 ± 50.8 to 84.1 ± 96.9 ml/100 ml/min, and for PDAC, from 45.8 ± 20.3 to 13.3 ± 18.7 ml/100 ml/min. The algorithm reduced noise impact, improving BF accuracy and CNR, with potential for lower-dose CT without compromising diagnostic quality.

**Supplementary Information:**

The online version contains supplementary material available at 10.1038/s41598-025-24482-x.

## Introduction

CT perfusion (CTp) is a non-invasive functional imaging technique that integrates physiological parameters and anatomical information of the tissue. It has become a powerful tool for early and accurate diagnosis of various diseases, including cardiovascular diseases^1,2^ such as coronary heart disease, stroke^3–5^, brain and body tumors, as well as (non-neoplastic) liver diseases^6,7^. By providing detailed insights into tissue physiology, CTp allows clinicians to assess blood flow (BF), blood volume (BV) and other critical information within tissues, which helps in better detection and characterization of pathological conditions^6,8–11^. However, despite its significant clinical use, the precision of CTp measurements can be severely compromised by image noise, an inherent limitation in CT imaging^12^.

Image noise, characterized by random variations in pixel intensity that do not correspond to the actual signal, leads to inaccuracies when quantifying tissue perfusion^13–15^. Errors in quantifying BF or BV within a tissue can result in misdiagnosis or inappropriate treatment response assessment, thus undermining the clinical effectiveness of CTp^13,14^. Therefore, mitigating image noise and improving image quality are vital for improving the accuracy of CTp measurements.

One approach to address these challenges is the use of digital perfusion phantoms (DPPs)^16,17^. These computer-generated models replicate the characteristics of biological tissues, allowing for controlled studies without ethical and practical constraints of patient data. Unlike physical phantoms, DPPs can simulate a broad range of conditions, are cost-effective, and are readily available. Moreover, they provide a known ground-truth for evaluation, unlike clinical datasets, where true perfusion values are unknown and which are prone to noise and artifacts^16,17^. This allows for customization of data specific to each study, avoiding problems such as radiation exposure to patients, image noise of unknown magnitude, or patient motion during CTp acquisitions as postulated by Manniesing et al.^16^.

In this study, a noise correction algorithm has been developed using DPPs to quantitatively evaluate and correct the influence of image noise on the accuracy of CTp BF measurements. Furthermore, the developed algorithm was subsequently applied retrospectively to an existing clinical dataset, providing an estimate of the precision of measured parameters and demonstrating its potential clinical applications.

## Results

Three patients were excluded due to final histopathological diagnoses other than pancreatic ductal adenocarcinoma (PDAC). Additionally, one patient was excluded as the lesion was missing in CT perfusion imaging, and five were excluded due to excessive breathing motion during dynamic data acquisition. As a result, final evaluation was conducted on 14 of the initial 23 patients. The demographic details of these 14 patients are provided in Table 1^18,19^.

**Table 1:** Demographic characteristics of the clinical dataset used for clinical evaluation in this study.

Demographic information	
Number of patients	14
Organ	Pancreas
Pathology	Pancreatic ductal adenocarcinoma (PDAC)
Median age (interquartile range)	63.1 (55–79 years)
*Sex* Female Male	8 6
*ROI sizes* (*Mean* (mm^2^) ± SD) PDAC Parenchyma	98.3 ± 45.9 70.3 ± 46.3

### Phantom evaluation

The mean ± SD values of ground-truth BF (GTBF), noise-impacted BF, and corrected BF obtained through the noise correction applied to DPPs are detailed in Table 2. For entire DPPs (whole pancreas), measured noise-impacted BF and noise-corrected BF were 140 ± 111 ml/100 ml/min and 131 ± 125 ml/100 ml/min, respectively, compared to GTBF of 131 ± 127 ml/100 ml/min. For DPP voxels assigned to non-neoplastic pancreatic parenchyma, GTBF, noise-impacted BF, and noise-corrected BF were 225 ± 120 ml/100 ml/min, 218 ± 112 ml/100 ml/min, and 224 ± 119 ml/100 ml/min, respectively. For voxels representing PDAC, these values were 37.5 ± 20.2 ml/100 ml/min, 62.1 ± 11.5 ml/100 ml/min, and 39.7 ± 21.9 ml/100 ml/min, respectively. Supplementary Fig. S1 shows visual examples of ground-truth, noise-impacted, and noise-corrected BF perfusion maps of a DPP.

**Table 2:** Mean ± SD blood flow (BF) values of the whole pancreatic region, non-neoplastic pancreatic parenchyma, and PDAC tissue using digital perfusion phantoms (DPPs). PDAC represent pancreatic ductal adenocarcinoma. GTBF and BFD represent the ground-truth BF measurements and the noise-impacted BF measurements, respectively. BFD_corr_(1)–BFD_corr_(7) represents the BF values through the noise correction algorithm with respective iterations. The Student’s t-test *p* value between GTBF and BFD values in each iteration indicates that the post-correction BF values are not significantly different from the GTBF for both non-neoplastic pancreatic parenchyma and PDAC. Contrast-to-noise ratio (CNR) shows an improvement from 2.52 to 2.61 between noise-impacted and noise-corrected BF maps.

Digital perfusion phantom evaluation									
Mean ± SD (ml/100 ml/min)	GTBF (Ground-truth BF)	BFD (Noise-impacted BF)	BFD_corr_(1)	BFD_corr_(2)	BFD_corr_(3)	BFD_corr_(4)	BFD_corr_(5)	BFD_corr_(6)	BFD_corr_(7)
Pancreas	131 ± 127	140 ± 111	137 ± 122	134 ± 125	133 ± 125	131 ± 124	132 ± 127	132 ± 126	131 ± 125
Non-neoplastic pancreatic parenchyma	225 ± 120	218 ± 112	225 ± 117	225 ± 119	225 ± 118	223 ± 117	225 ± 120	225 ± 120	224 ± 119
	T-test (*p* value)	0.000	0.158	0.456	0.674	0.814	0.922	0.867	0.796
PDAC	37.5 ± 20.2	62.1 ± 11.5	48.1 ± 16.8	43.4 ± 19.2	40.9 ± 20.9	39.5 ± 22.7	38.3 ± 22.5	38.9 ± 21.2	39.7 ± 21.9
	T-test (*p* value)	0.886	0.987	0.987	0.986	0.971	0.989	0.996	0.984
CNR	2.66	2.52	2.64	2.63	2.65	2.63	2.62	2.64	2.61

**Fig. 1:**
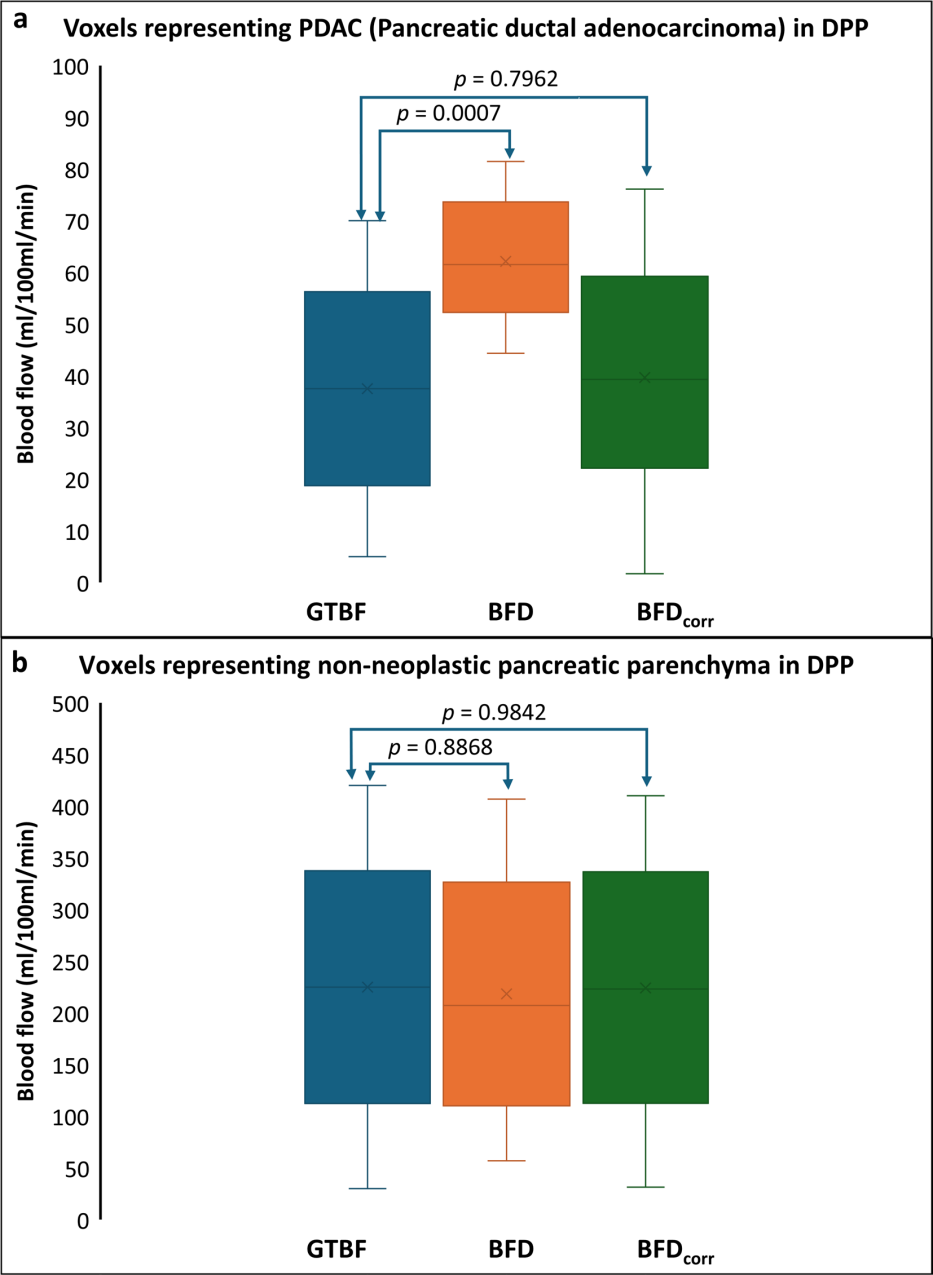
Box plot showing significance levels (*p* values calculated using Student’s t-test) comparing GTBF, BFD, and BFD_corr_ measurements for (**a**) voxels corresponding to the PDAC region, and (**b**) voxels representing the non-neoplastic pancreatic parenchyma tissue in the DPPs. GTBF = ground-truth blood Flow, BFD = uncorrected blood flow, *BFD*_*corr*_  = corrected blood flow, *PDAC* = pancreatic ductal adenocarcinoma, *DPP* = digital perfusion phantom.

Linear regression analysis showed a strong correlation between input blood flow (BFD) and GTBF (R^2^ = 0.9923), which further improved after correction between corrected BF value (BFD_corr_) and GTBF (R^2^ = 0.9989). Box-plots displayed in Fig. 1 provide a comparative analysis of GTBF, BFD and BFD_corr_ measurements derived from DPPs. The Student’s t-test indicated no statistically significant difference between GTBF and BFD_corr_, post-correction.

**Fig. 2:**
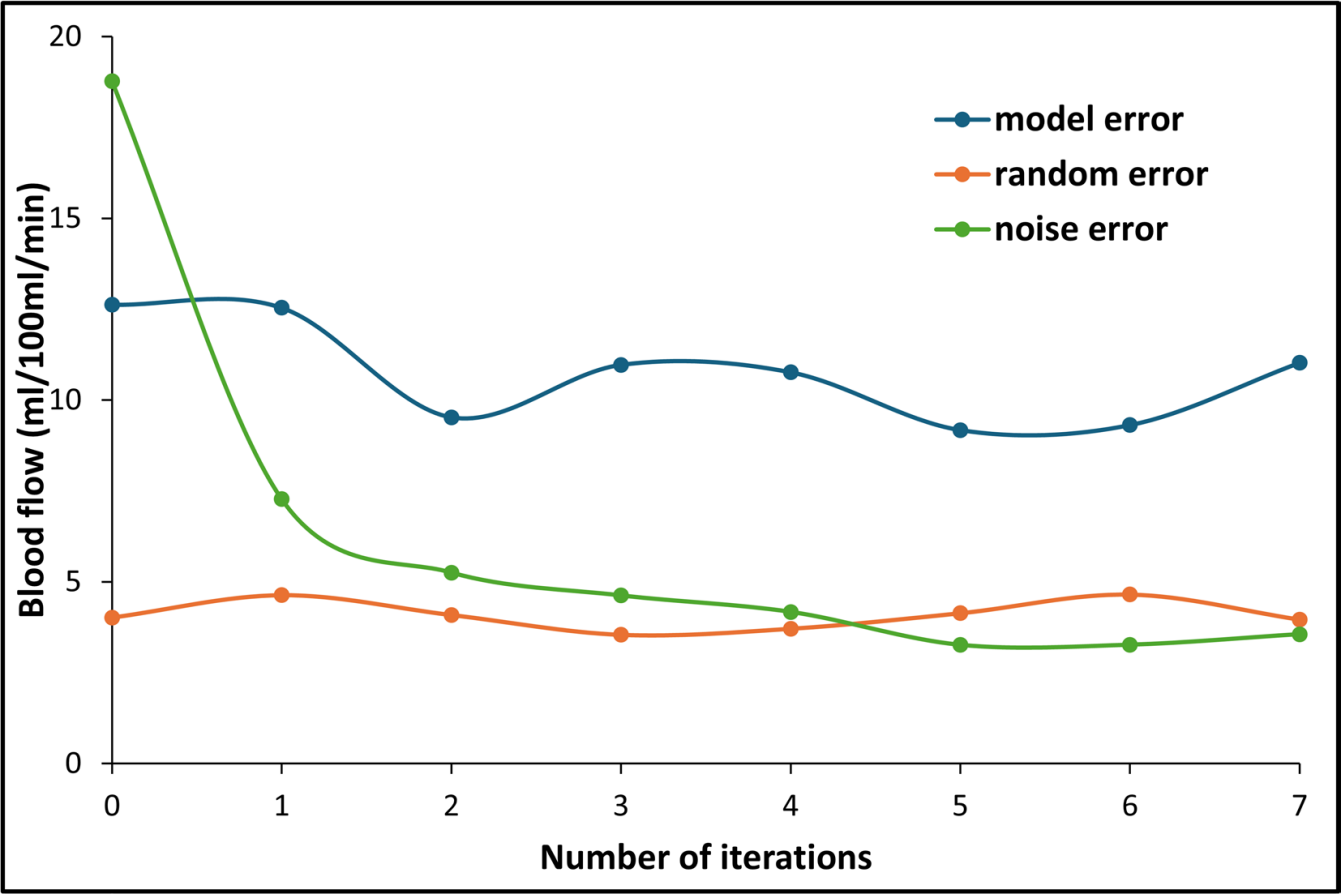
Noise error, model error, and random error curves across iterations of the noise correction algorithm.

After noise correction, the noise error decreased from 18.7 to 3.56 ml/100 ml/min (see Table 3 and Fig. 2). Additionally, the model error decreased from 12.6 to 11.0 ml/100 ml/min, while the random error showed a slight change from 4.02 to 3.96 ml/100 ml/min. The Student’s t-test showed a significant difference between PDAC and non-neoplastic pancreatic parenchyma for noise-impacted and noise-corrected BF values (*p* < 0.05). Noise correction also improved contrast-to-noise ratio (CNR) from 2.52 to 2.61, as detailed in Table 2.

**Table 3:** The noise error, model error, and random error for each iteration and noise-impacted BF measurements. BFD represents the noise-impacted BF and BFD_corr_(1)–BFD_corr_(7) represents BF measurements with each iteration through the noise correction algorithm.

Errors observed in digital perfusion phantom evaluation								
Errors	BFD (Noise-impacted BF)	BFD_corr_(1)	BFD_corr_(2)	BFD_corr_(3)	BFD_corr_(4)	BFD_corr_(5)	BFD_corr_(6)	BFD_corr_(7)
Noise error	18.7	7.28	5.25	4.63	4.17	3.27	3.27	3.56
Model error	12.6	12.5	9.53	10.9	10.7	9.18	9.31	11.0
Random error	4.02	4.64	4.09	3.54	3.71	4.14	4.65	3.96

**Fig. 3:**
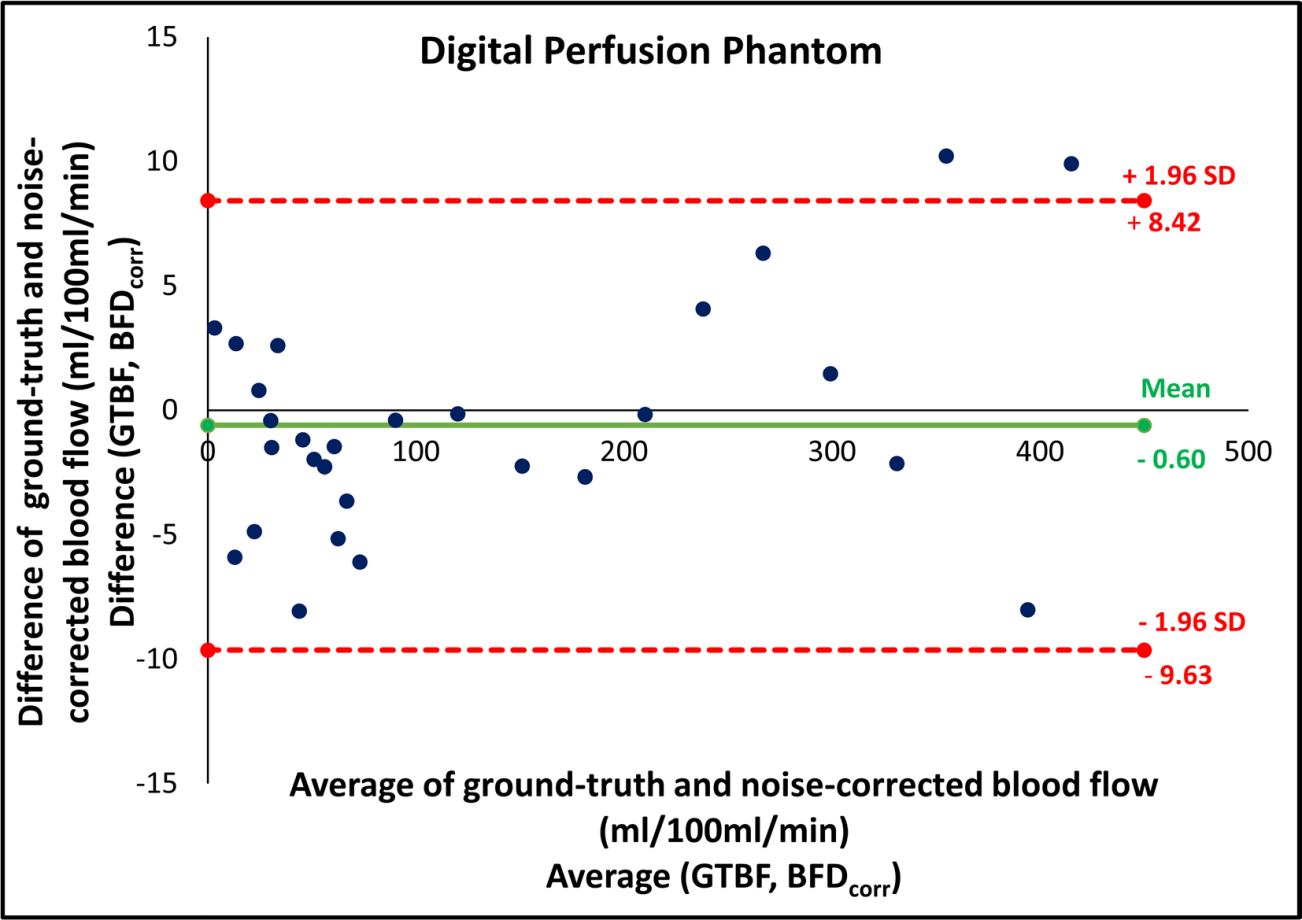
Bland–Altman plots for digital perfusion phantom (DPP) including the limits of agreement, showing a comparison between ground-truth (GTBF) and noise-corrected blood flow (BFD_corr_) measurements.

Figure 3 presents Bland–Altman analysis comparing GTBF with BFD_corr_ for DPP. The limits of agreement between GTBF and BFD_corr_ were − 9.63 to 8.42 ml/100 ml/min.

### Clinical evaluation

Table 4 presents mean ± SD values of noise-impacted BF, and corrected BF obtained using the noise correction algorithm in the clinical dataset. For the entire pancreatic region, measured noise-impacted BF was 97.1 ± 64.2 ml/100 ml/min, while noise-corrected BF was 84.1 ± 96.9 ml/100 ml/min. For non-neoplastic parenchyma tissue, noise-impacted BF was 148 ± 50.8 ml/100 ml/min, and corrected BF was 155 ± 91.5 ml/100 ml/min. For PDAC, noise-impacted BF was 45.8 ± 20.3 ml/100 ml/min, and corrected BF was 13.3 ± 18.7 ml/100 ml/min. The Student’s t-test showed a significant difference between PDAC and non-neoplastic pancreatic parenchyma for noise-impacted and noise-corrected BF values (*p* < 0.05). After noise correction, CNR changed from 2.88 to 2.57 when comparing noise-impacted and noise-corrected BF maps.

**Table 4:** Mean ± SD blood flow (BF) values of pancreatic region, non-neoplastic pancreatic parenchyma, and PDAC tissue in the clinical dataset. PDAC represent pancreatic ductal adenocarcinoma. BFD represents the noise-impacted BF measurements and BFD_corr_(1)–BFD_corr_(7) represents the BF values through the noise correction algorithm with respective iterations. The contrast-to-noise ratio (CNR) does not show any improvement, but lies in a reasonable range.

Clinical evaluation								
Mean ± SD (ml/100 ml/min)	BFD (Noise-impacted BF)	BFD_corr_(1)	BFD_corr_(2)	BFD_corr_(3)	BFD_corr_(4)	BFD_corr_(5)	BFD_corr_(6)	BFD_corr_(7)
Pancreas	97.1 ± 64.2	86.9 ± 79.5	83.5 ± 85.1	84.3 ± 89.0	84.5 ± 91.5	82.5 ± 92.2	83.6 ± 94.2	84.1 ± 96.9
Non-neoplastic pancreatic parenchyma	148 ± 50.8	147 ± 67.9	147 ± 75.9	149 ± 81.1	152 ± 83.4	151 ± 85.1	153 ± 87.5	155 ± 91.5
PDAC	45.8 ± 20.3	26.6 ± 27.7	20.0 ± 25.8	18.7 ± 25.7	16.4 ± 22.8	14.0 ± 19.2	13.8 ± 18.8	13.3 ± 18.7
CNR	2.88	2.52	2.50	2.46	2.56	2.63	2.63	2.57

**Fig. 4:**
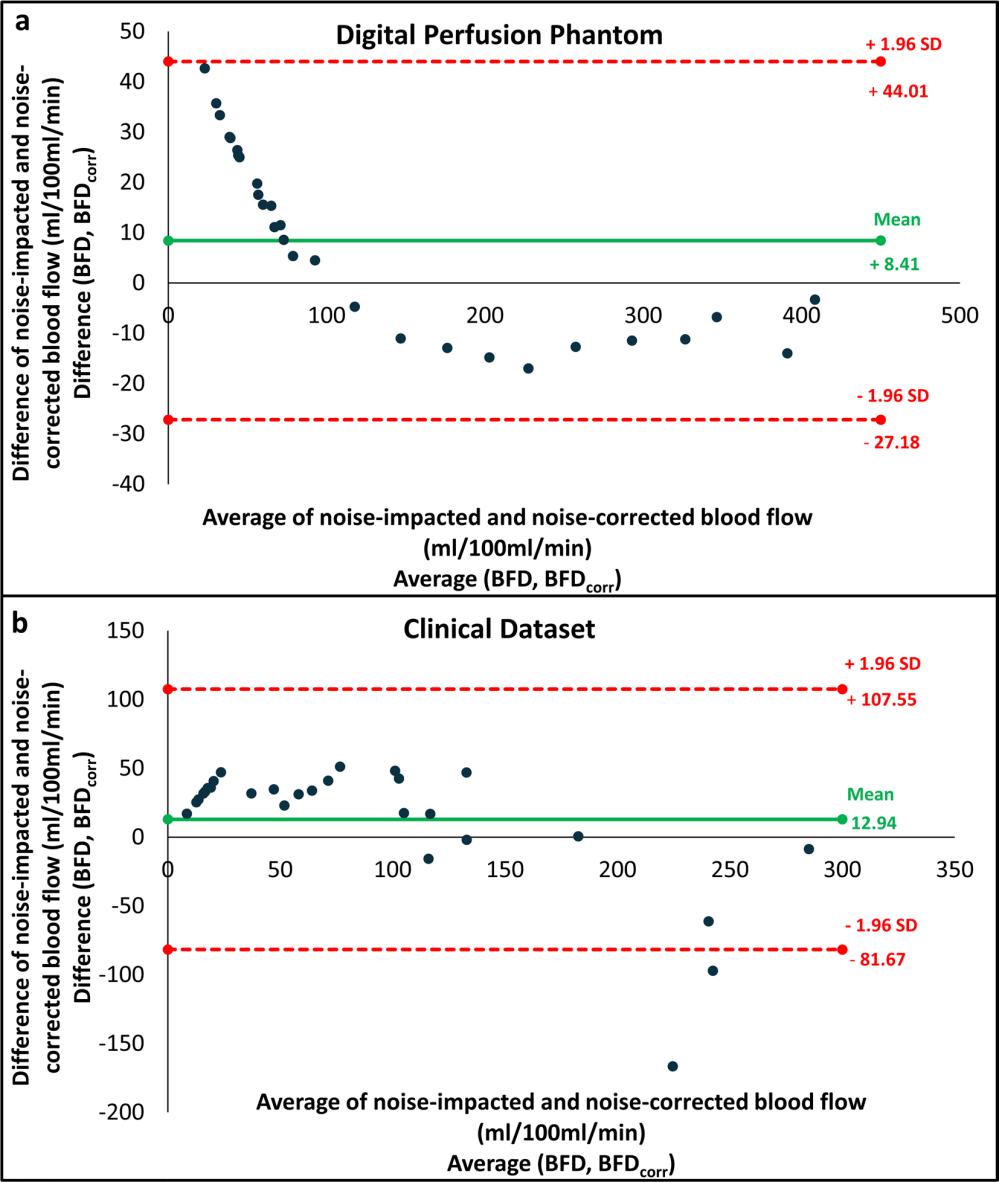
Bland–Altman plot including the limits of agreement, showing a comparison between the noise-impacted (BFD) and noise-corrected blood flow (BFD_corr_) measurements in the (**a**) Digital Perfusion Phantom and (**b**) the clinical dataset.

Figure 4 displays Bland–Altman analysis comparing BFD and BFD_corr_ measurements for both DPP and clinical dataset. For DPP, limits of agreement were − 27.1 to 44 ml/100 ml/min, while for clinical dataset, limits ranged from − 81.6 to 107 ml/100 ml. As part of noise correction process, all BF values below approximately 50 ml/100 ml/min resulted in 0 ml/100 ml/min. BF values around 100 ml/100 ml/min were mostly unaffected by the added noise, i.e. close to zero in Bland–Altman plot.

## Discussion

Previous studies have shown that image noise negatively impacts the accuracy of CTp measurements, leading to complications in interpreting perfusion parameters and potentially compromising clinical decisions^13,14^. In this study, we developed a noise correction algorithm using DPPs to quantitatively assess and correct the impact of image noise on CTp BF measurements. The algorithm was evaluated using both simulated and clinical datasets, demonstrating its effectiveness in reducing inaccuracies in perfusion measurements.

The results show that the algorithm can reduce inaccuracies in perfusion measurements caused by image noise. Student’s t-test suggests that errors compared to ground-truth were no longer significant after correction. The noise error decreased from 18.7 to 3.56 ml/100 ml/min in the phantom and falls below or is almost at the same level of the model error and the random error after a few iterations (see Fig. 2). This indicates that the algorithm effectively accounts for variability in BF measurements caused by image noise, as the remaining deviation can be attributed to random effects and that the algorithm has converged to a stable solution. Increasing the number of tissue attenuation curves (TACs) could further decrease noise error at the cost of increased processing time.

Bland–Altman analysis showed a higher level of agreement between uncorrected BF and noise-corrected BF for DPP (refer to Fig. 4), compared to a clinical dataset, indicating a greater impact of noise-correction on BF in the clinical dataset. This can be due to the reason that fixed parameters such as BV, mean transit time (MTT), and flow extraction product (EF), derived from Mayer’s study^18^, were used for TAC simulations. These parameters were already impacted by image noise and were kept constant across iterations to simplify the correction process as the algorithm corrected only BF values while other physiological parameters influencing perfusion, such as BV and MTT, were not included in the corrections, which may have reduced the accuracy of noise correction in clinical data. Additionally, the higher SD (refer to Table 4) observed after correction in the clinical dataset might have resulted from variability in individual patient responses, as some patients exhibited higher BF values than others and their impact might be amplified by the limited sample size.

Despite the improvements in BF estimation, a slight reduction in CNR was observed in the clinical dataset following noise correction. This may be due to fixed perfusion parameters used during the correction process, and technical constraints of the clinical perfusion software (syngo.via) that allowed calculation of only mean values from pre-defined ROIs. As a result, the CNR values represent collective averages across patients rather than individualized or voxel-wise assessments. Future work for patient specific analysis may offer deeper insights into changes in CNR.

The algorithm improved BF measurements for non-neoplastic pancreatic parenchyma and PDAC, with noise-corrected values differing by 36% in PDAC and only 2.6% in non-neoplastic pancreatic parenchyma, as shown in Tables 2, 4 and Fig. 1. This difference can be attributed to the fact that BF in parenchyma has higher values, which are less affected by image noise and thus show limited change after correction. In contrast, PDAC, where BF is lower, is more prone to underestimation and benefits more substantially from noise correction (see Fig. 4). These findings indicate increased vulnerability of PDAC to image noise, and show the importance of precise evaluations in managing PDAC. It should be noted that similar effects might be observed in other organs and pathologies, prompting a broader consideration of these outcomes beyond the scope of pancreatic studies to reduce potential inaccuracies in perfusion measurements.

Comparing our findings with existing studies on noise correction in perfusion imaging such as İnal et al.^15^, Mendrik et al.^21^, and Kulvait et al.^20^, the developed algorithm provides a unique contribution to the field by effectively mitigating the impact of image noise on quantitative BF measurements. This study, in particular, demonstrates that image noise can lead to an underestimation of perfusion values when BF is high, as seen in DPP analysis. The clinical implications of these improved measurements are significant, with the potential to enhance diagnostic accuracy and better treatment planning for pancreatic patients. In addition, the algorithm’s robustness in correcting noise effects on perfusion measurements suggests that it could be applied even to lower quality or lower dose CT acquisitions, potentially enabling perfusion data acquisition with reduced radiation exposure while maintaining diagnostic quality.

A key limitation is the absence of ground-truth in the clinical dataset, along with the occurrence of BF values approaching zero after correction in certain cases, where it is unclear whether the corrected near-zero BF values represent underestimation or true physiological values. While a clear separation between very low BF in tumor tissue and higher BF in normal parenchyma may help in differentiation in diagnostic imaging, misinterpreting underestimated BF values could potentially lead to false conclusions in treatment monitoring. Additionally, the algorithm focused exclusively on BF, without addressing other perfusion parameters such as BV, EF, or MTT, which can also be affected by image noise. Noise in AIFs (arterial input function) was not considered, which, despite being averaged over regions of interest to reduce noise, can still affect CTp measurements. However, due to limitations in commercially available CTp software, it was not feasible to evaluate AIF noise effects in the same manner as TACs.

All evaluations in this study were performed using a commercial deconvolution model (syngo.via, Siemens Healthineers) with fixed reconstruction parameters such as slice thickness, reconstruction kernel, and matrix size selected to reflect standard clinical CTp practice. These parameters are known to influence image noise characteristics, which can, in turn, affect both the accuracy of perfusion measurements and the performance of noise correction algorithm. While our results demonstrate robust algorithmic performance under these conditions, its behavior under alternative reconstruction settings (e.g., sharper kernels or thinner slices) or at different levels of image noise remains to be investigated. In particular, the current study used a single Gaussian noise level (SD = 25), derived from a clinical dataset^18^ and supported by a recent meta-analysis of pancreatic CT perfusion studies^22^. Although this value reflects conditions frequently encountered in clinical pancreatic CTp, noise characteristics can differ across scanners and imaging protocols. Future work should therefore evaluate algorithm performance across multiple noise levels to ensure broader applicability and generalizability.

Importantly, perfusion values in this study were derived from averaged values within predefined ROIs rather than on a voxel-wise basis. This spatial averaging may have helped to minimize the variability introduced by differences in image reconstruction. However, the generalizability of our findings across different sites, and acquisition protocol remains an essential direction for future research.

We also acknowledge that this study’s evaluations were based on the commercial implementation of a deconvolution model from a single vendor. While the findings are expected to be broadly applicable, caution is needed when generalizing these results to other models and vendors.

In conclusion, the noise correction algorithm effectively reduces noise error i.e., absolute difference between corrected BF measurements and ground-truth values, demonstrating its potential in correcting the impact of image noise on BF measurements for PDAC patients. With further refinement, this algorithm could improve comparability across patients, imaging centers, and equipment vendors, ultimately improving diagnostic accuracy and treatment planning, and potentially facilitating perfusion data acquisition with reduced radiation exposure.

## Methods

### Dataset

The study protocol was approved by the local ethics committee of Heidelberg University Hospital and was conducted in accordance with ethical standards of the World Medical Association (Declaration of Helsinki). All subjects provided written informed consent before image data acquisition. The retrospective analysis included data from 59 PDAC patients, as detailed by Klauss et al.^23^, Fritz et al.^9^ and Mayer et al. ^18^. The AIF was derived from 59 patients, while 23 patients from a recent CTp study^18^ were selected for clinical evaluation of the developed algorithm.

### Inclusion and exclusion criteria

Patients with a pancreatic mass suspicious of PDAC identified in prior clinical examinations were included. Exclusion criteria were prior treatment for PDAC, final histopathological diagnosis other than PDAC, hyperthyroidism, reduced kidney function, hypersensitivity to iodinated contrast agents, inability to maintain required breathing technique during image acquisition, or denial of consent, as per Mayer et al.^18^.

### Data acquisition

Dynamic contrast-enhanced abdominal CT acquisitions were performed using a dual-source, dual-energy CT scanner (SOMATOM Definition Flash; Siemens Healthineers, Germany). Preceding dynamic acquisition, 80 ml non-ionic iodinated contrast agent (Ultravist 370; Schering, Germany) was injected intravenously at a rate of 5.0 ml/s, followed by a 40 ml bolus of saline solution (NaCl). The acquisition started 13 s after the initiation of contrast agent injection and included 34 axial acquisitions at 1.5 s intervals over 51 s (0.5 s acquisition time, 1.5 s cycle time) with tube voltage of 80 kVp/140 kVp and automated tube current modulation (270 mAs/104 mAs). The scan coverage was 19.2 mm. Image reconstruction was performed using a soft tissue kernel B30f and 0.6 mm slice thickness.

### Noise correction algorithm

A noise correction algorithm was developed to improve the accuracy of CTp BF measurements affected by image noise. The algorithm was implemented in MATLAB R2022a (MathWorks; USA) and evaluated using a commercially available workstation (syngo.via Body perfusion VB10B; Siemens Healthineers)^9,24^. This method allows for the correction of noise effects in pancreatic perfusion measurements, producing an estimate of GTBF for BFD and SD. A detailed explanation of algorithm development and noise correction of BF measurements is as follows:

*Step-1* AIF Averaging

**Fig. 5:**
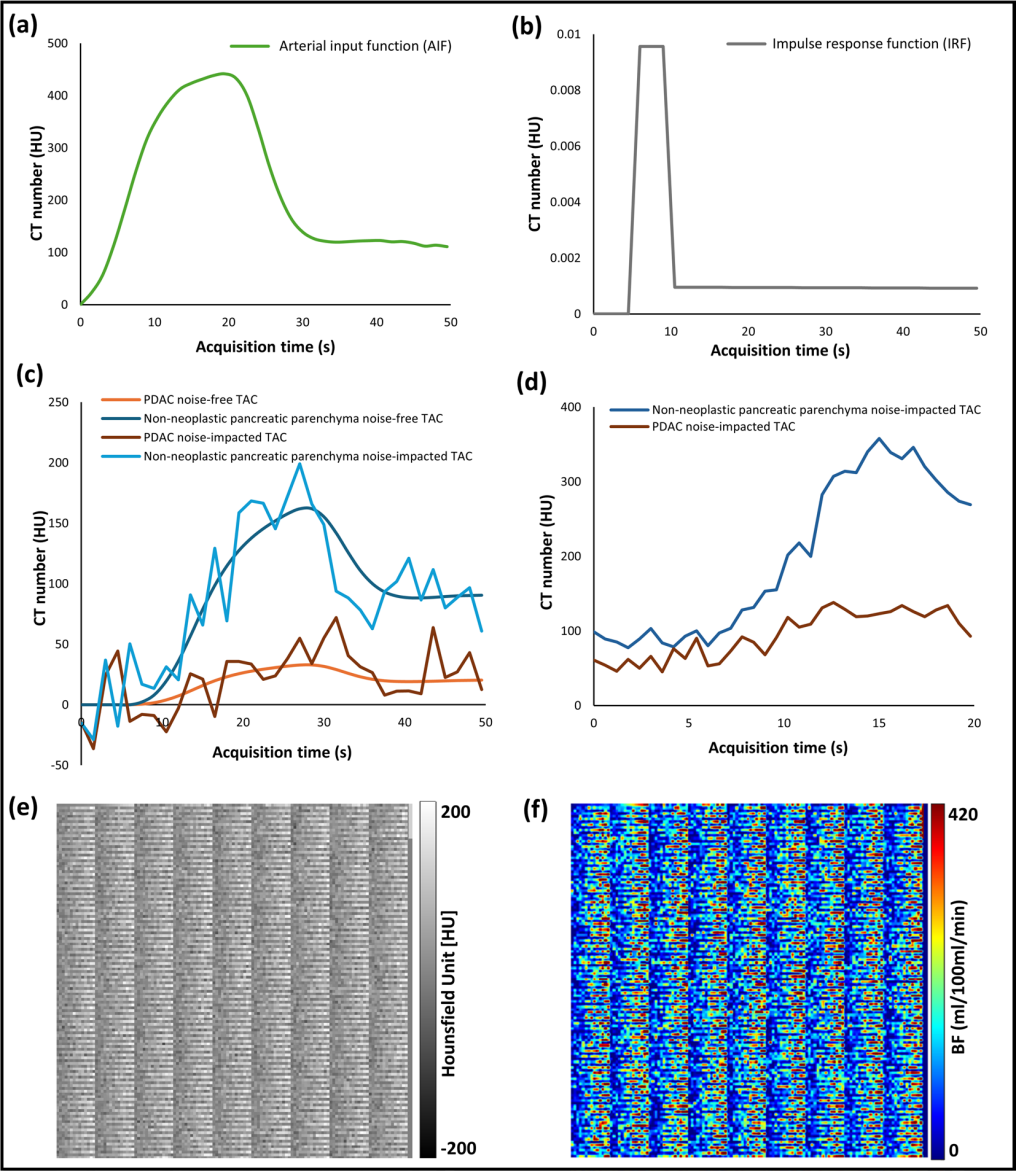
A detailed explanation of the output obtained at each step during development of the noise correction algorithm. (**a**) An arterial input function (AIF) of a digital perfusion phantom (DPP) with zero baseline and temporal sampling rate of 1.5 s, generated by averaging the AIFs from a cohort of 59 pancreatic ductal adenocarcinoma (PDAC) patients. (**b**) An example of the simulated impulse response function (IRF) of a DPP. (**c**) An example of noise-free and noise-added tissue attenuation curves (TACs) for a DPP generated for both tissue types, PDAC and non-neoplastic pancreatic parenchyma. (**d**) An example of TACs from non-neoplastic pancreatic parenchyma and PDAC regions of a PDAC patient for comparison with the simulated TACs. (**e**) An example of an image from the DICOM series of a DPP made up of 16,128 (28 sets of ground-truth values * 576 random noise samples) TACs. The top right corner of the phantom image represents the AIF. (**f**) An example of a blood flow (BF) map obtained from a DPP using commercially available CT perfusion software (syngo.via Body perfusion VB10B, Siemens Healthineers).

Averaged AIF was obtained from 59 PDAC patients, shown in Fig. 5a.

*Step-2* IRF (Impulse Response Function) Generation

IRF was generated using an adiabatic approximation to the tissue homogeneity model ^25,26^, as described by the equation below:

1 $$IRF\left( t \right) = \left\{ {\begin{array}{*{20}c} {0,} \\ {BF,} \\ {EF \cdot e^{{ - EF\frac{t - TTD}{{V_{e} }}}} ,} \\ \end{array} \begin{array}{*{20}c} {t < TTS} \\ {TTS < t < TTD} \\ {t > TTD} \\ \end{array} } \right.$$

where EF is the flow extraction product, TTS is the time taken by the contrast agent to reach the tissue, and TTD is the total time taken by the contrast agent from start of injection to drain i.e. TTD = TTS + MTT.

V_e_ (volume in the extracellular extravascular space)^6^ was derived using the equation below, where ground-truth BV (GTBV) was calculated by multiplying GTBF with MTT:

2 $$V_{e} = 1 - \frac{GTBV}{{100}} - 0.02$$

The constant 0.02 was experimentally determined based on prior work (Klotz et al.^27^) to avoid numerical instabilities depending on GTBV values. Figure 5b illustrates an example of generated IRF.

*Step-3* TAC Generation

TACs were generated by convolving AIF with IRF, shown in Fig. 5c.

3 $$TAC = AIF \otimes IRF$$

The temporal sampling of simulated TACs was chosen to match the sampling rates used in clinical datasets (i.e. 1.5 s).

*Step-4* Noise Introduction

To simulate the impact of image noise on perfusion measurements, Gaussian noise was added to TACs. For each independent TAC, 2 × 576 noise-impacted TACs were generated, forming two distinct phantoms of 576 noisy TACs each, thereby allowing for the estimation of the random error (see below). The number of samples per phantom (576) was selected to ensure sufficient number of data points for robust statistical analysis and iterative optimization during noise correction. A mean of 0 and SD of 25 HU were used to simulate noise for the phantom dataset, while patient-derived noise was used for the clinical dataset. Figure 5c illustrates an example of this simulated noisy TACs, while Fig. 5d presents an example of TACs from non-neoplastic pancreatic parenchyma and PDAC regions of a PDAC patient, demonstrating the comparison with simulated TACs.

*Step-5* Digital Image Representation

Noise-impacted TACs were represented as digital images stored in DICOM format, with pixel regions designated for TACs and AIF values. An example image from this dynamic DICOM series is presented in Fig. 5e, which displays a single time frame from the dynamic perfusion acquisition for visual reference. Although only one frame is shown, the full dynamic series was used for BF calculation.

*Step-6* Processing of Digital Images

Digital images were processed using a deconvolution model on a CTp workstation (syngo.via Body Perfusion; Siemens Healthineers) to generate BF maps, which were exported in DICOM format for further analysis. An example BF map is shown in Fig. 5f.

*Step-7* Calculation of BF Values

Quantitative BF values were extracted from the BF maps for each noise-impacted TAC. BF values (BF_1_ and BF_2_), were calculated for each of the 2 × 576 noisy TACs, and averages of the 2 × 576 BF measurements (BF_avg_) were computed for analysis.

*Step-8* Noise Correction

In this step, original BF measurements (BFD) were compared to the average of two noise-affected samples (BF_1_ and BF_2_), denoted as BF_avg_. The difference between BFD and BF_avg_ was used to adjust and refine the measurements, calculating BFD_corr_:

4 $$BFD_{corr} = BFD + \left( {BFD - BF_{avg} } \right)$$

Any BFD_corr_ values below zero were truncated, as they have no physiological relevance.

This process was repeated iteratively by starting the whole process from Step-2 with the corrected values BFD_corr_ from previous iteration serving as input BFD (BFD_in_) for the next iteration, recalculating corrected IRF, and generating new TACs with added Gaussian noise. This iterative process was continued until the difference between BFD and BF_avg_ approached zero, indicating successful noise correction. The underlying idea of this algorithm was that observing the shift in BF measurements caused by adding synthetic noise to a TAC allows to estimate GTBF without noise by subtracting that shift from the measurement.

### Phantom evaluation

The algorithm was evaluated using DPPs generated according to steps 1–5 described above, with well-defined perfusion parameters, considered ground-truth parameters, derived from our previously published meta-analysis^22^ to simulate pancreatic CTp. The GTBF ranged from 5 to 420 ml/100 ml/min, encompassing a total of 28 sets—14 representing non-neoplastic pancreatic parenchyma (30 to 420 ml/100 ml/min) and 14 for PDAC (5 to 70 ml/100 ml/min).

To maintain consistency in simulation, fixed values were used for extraction fraction (E), TTS, and MTT, set at 0.1, 5 s, and 5 s, respectively. This resulted in TTD of 10 s. These values were selected based on ranges reported in our previously published meta-analysis study on pancreatic perfusion^22^. Additionally, a temporal sampling of 1.5 s intervals was chosen for simulated DPPs to reflect sampling rates typically observed in clinical datasets.

To simulate noise, 2 × 576 random samples of Gaussian noise (mean = 0 HU, SD = 25 HU) were added to DPPs to obtain noise-impacted BF measurements. Afterwards, the noise correction algorithm was applied iteratively to correct these noise-impacted BF measurements. The error between GTBF values and corrected measurements was monitored, stopping when the error approached a sufficiently low threshold after seven iterations, indicating that the algorithm was effectively correcting perfusion measurements.

**Fig. 6:**
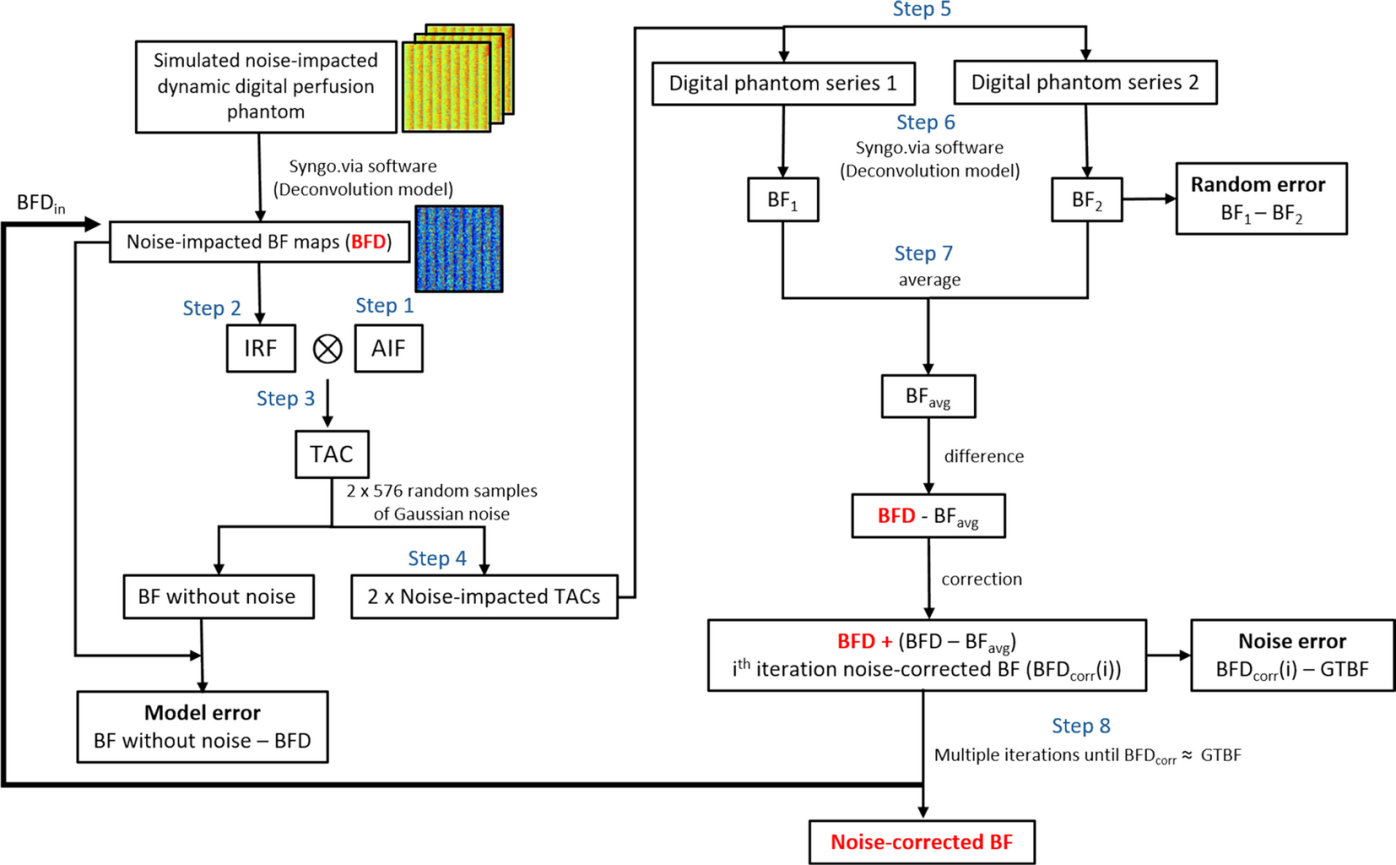
A flow chart illustrating the complete noise correction process for blood flow (BF) measurements, including evaluation using digital perfusion phantom (DPP) (starting from the first block) and evaluation using a clinical dataset (starting from the second block). For the DPP analysis, each GTBF value is simulated using two independent sets of 576 noise-impacted TACs, resulting in BF1 and BF2 estimates for random error calculation. This process is repeated for 28 GTBF values, totaling 16,128 TACs. For the clinical dataset, patient BF values calculated using the deconvolution model from Mayer’s study^18^ were used as input for the noise-impacted BF maps. BFD represents the noise-impacted BF measurements, which need to be corrected. IRF is the impulse response function, AIF is the arterial input function, TAC represents the tissue attenuation curve, and GTBF is the ground-truth blood flow. BFD_corr_(i) represents the noise-corrected BF measurement for the i^th^ iteration. The random error and model error calculations are also shown in the flow chart. This iterative process for DPP continues until BFD_corr_ aligns with GTBF or until the error between GTBF and corrected measurements is minimized to an acceptable threshold.

For each iteration, absolute differences between BFD_corr_ and GTBF were calculated, referred to as “noise error”, i.e. error caused by the added noise. The mean noise error was determined across all iterations. Additionally, model and random errors were calculated for each iteration. Random error was determined by computing the absolute difference between BF_1_ and BF_2_ at each iteration, i.e. difference between two random samples of 576 TACs, as illustrated in Fig. 6. Model error was calculated as the absolute difference between BFD_in_ and that BF with no noise, i.e. error of simulation and measurement in the absence of noise (refer to Fig. 6).

### Clinical evaluation

The algorithm was evaluated using a clinical dataset from a previous CTp study^18^ comprising 23 patients with PDAC. Noise-impacted BF measurements, calculated using a deconvolution model from Mayer et al.^18^, were used as the input for the correction process. Image noise quantified by ROI (region of interest) measurement on image data from Mayer et al.^18^, was used to simulate noise in the clinical dataset. Fixed values for parameters BV, MTT, and EF, also obtained from Mayer et al.^18^, were used for TAC simulations. These values, influenced by image noise, were kept constant across iterations to simplify the correction process. The algorithm was applied to both non-neoplastic pancreatic parenchyma and PDAC to obtain noise-corrected BF measurements for both the tissue regions.

### Statistical analysis

Statistical analysis was performed using Excel 2016 (Microsoft Corp.). To assess the relationship between BFD and BFD_corr_ measurements, linear regression analysis and Bland–Altman analysis was performed. A student’s t-test was used to compare BF values for non-neoplastic pancreatic parenchyma and PDAC and to analyze differences between GTBF and BFD_corr_ values separately for voxels representing non-neoplastic pancreatic parenchyma and PDAC, respectively. Additionally, CNR, a key parameter for assessing image quality in relation to noise, was calculated at each iteration. The equation used for CNR calculation is elaborated below:

5 $$CNR = \frac{Contrast}{{Noise}} = \frac{{\frac{{\sum \left( {BFD_{corr} \left( {parenchyma} \right) - BFD_{corr} \left( {carcinoma} \right)} \right)}}{Number\ of\ datasets}}}{{\frac{{\sigma_{{BFD_{corr} \left( {parenchyma} \right)}} + \sigma_{{BFD_{corr} \left( {carcinoma} \right)}} }}{2}}}$$

where σ represents the SD of corrected BF values within the parenchyma and carcinoma regions, and the total number of datasets were 14 for DPPs and 14 for patient datasets.

## Supplementary Information

Below is the link to the electronic supplementary material.

Supplementary Material 1

## Data Availability

The datasets analyzed during the current study are not publicly available due to ethical reasons but are available from the corresponding author on reasonable request.
