# Correction: Model based noise correction enhances the accuracy of pancreatic CT perfusion blood flow measurements

**DOI:** 10.1038/s41598-026-36173-2

**Published:** 2026-02-05

**Authors:** Neha Vats, Philipp Mayer, Franziska Kortes, Miriam Klauß, Lars Grenacher, Hans-Ulrich Kauczor, Wolfram Stiller, Stephan Skornitzke

**Affiliations:** 1 Clinic for Diagnostic and Interventional Radiology (DIR), Heidelberg University Hospital, Heidelberg, Germany; 2 Department of Neuroimaging, Central Institute of Mental Health, Medical Faculty Mannheim, Heidelberg University, Mannheim, Germany; 3 Radiology Rhein-Neckar, Schwetzingen, Germany; 4 MVZ Radiological and Nuclear Medicine Diagnostic Center, Conradia Radiology Munich, Munich, Germany; 5 Philips Healthcare, Hamburg, Germany

Correction to: *Scientific Reports* 10.1038/s41598-025-24482-x, published online 23 October 2025

The original version of this Article contained an error in the order of the Figures. Figure 5 was published as Figure 1, Figure 6 was published as Figure 2, Figure 1 was published as Figure 3, Figure 2 was published as Figure 4, Figure 3 was published as Figure 5, and Figure 4 was published as Figure 6. The Figure legends were correct.

The original Figures 1, 2, 3, 4, 5, 6 and accompanying legends appear below.

**Fig. 1:**
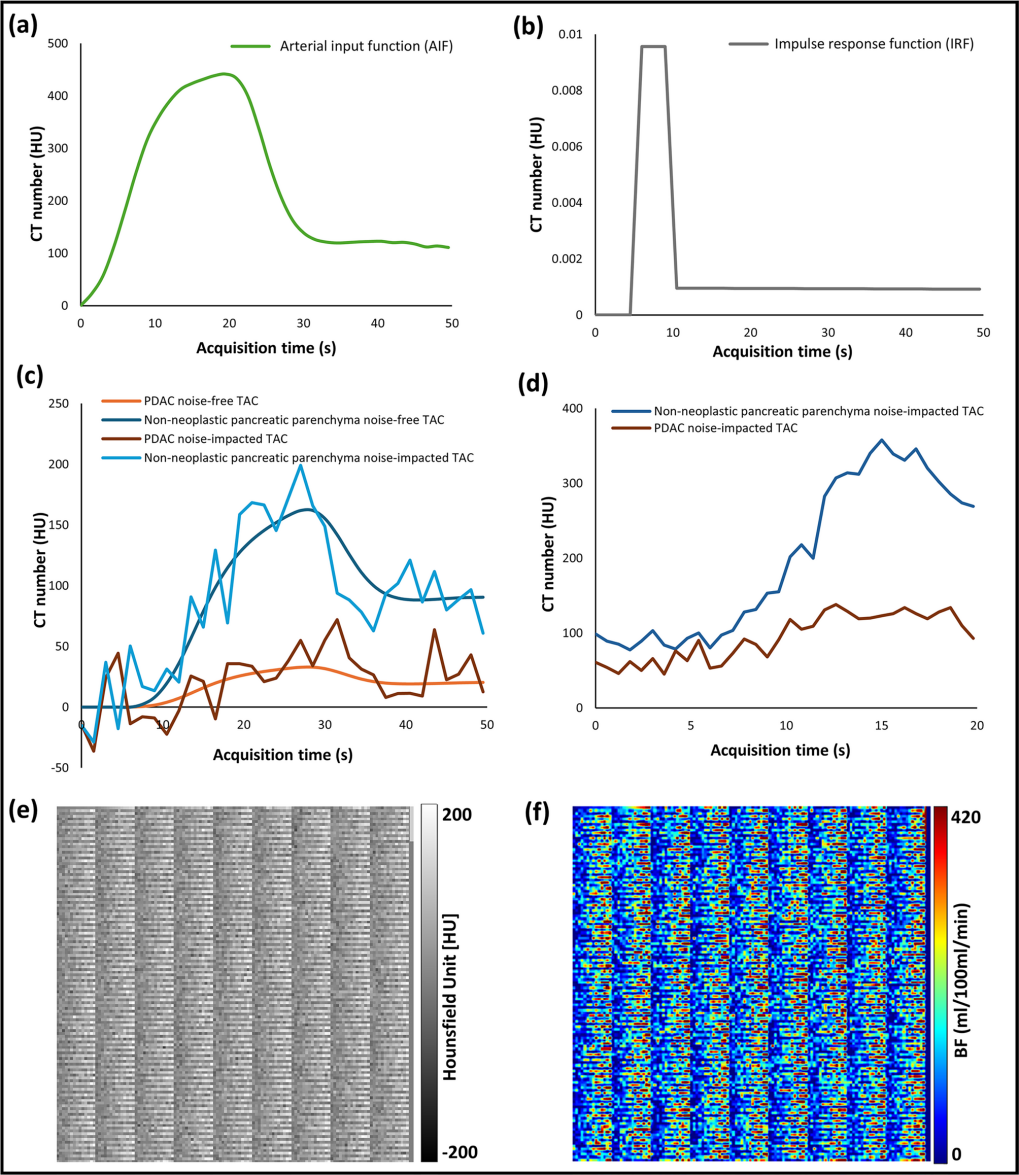
Box plot showing significance levels (*p* values calculated using Student’s t-test) comparing GTBF, BFD, and BFD_corr_ measurements for (**a**) voxels corresponding to the PDAC region, and (**b**) voxels representing the non-neoplastic pancreatic parenchyma tissue in the DPPs. GTBF = ground-truth blood Flow, BFD = uncorrected blood flow, *BFD*_*corr*_  = corrected blood flow, *PDAC* = pancreatic ductal adenocarcinoma, *DPP* = digital perfusion phantom.

**Fig. 2:**
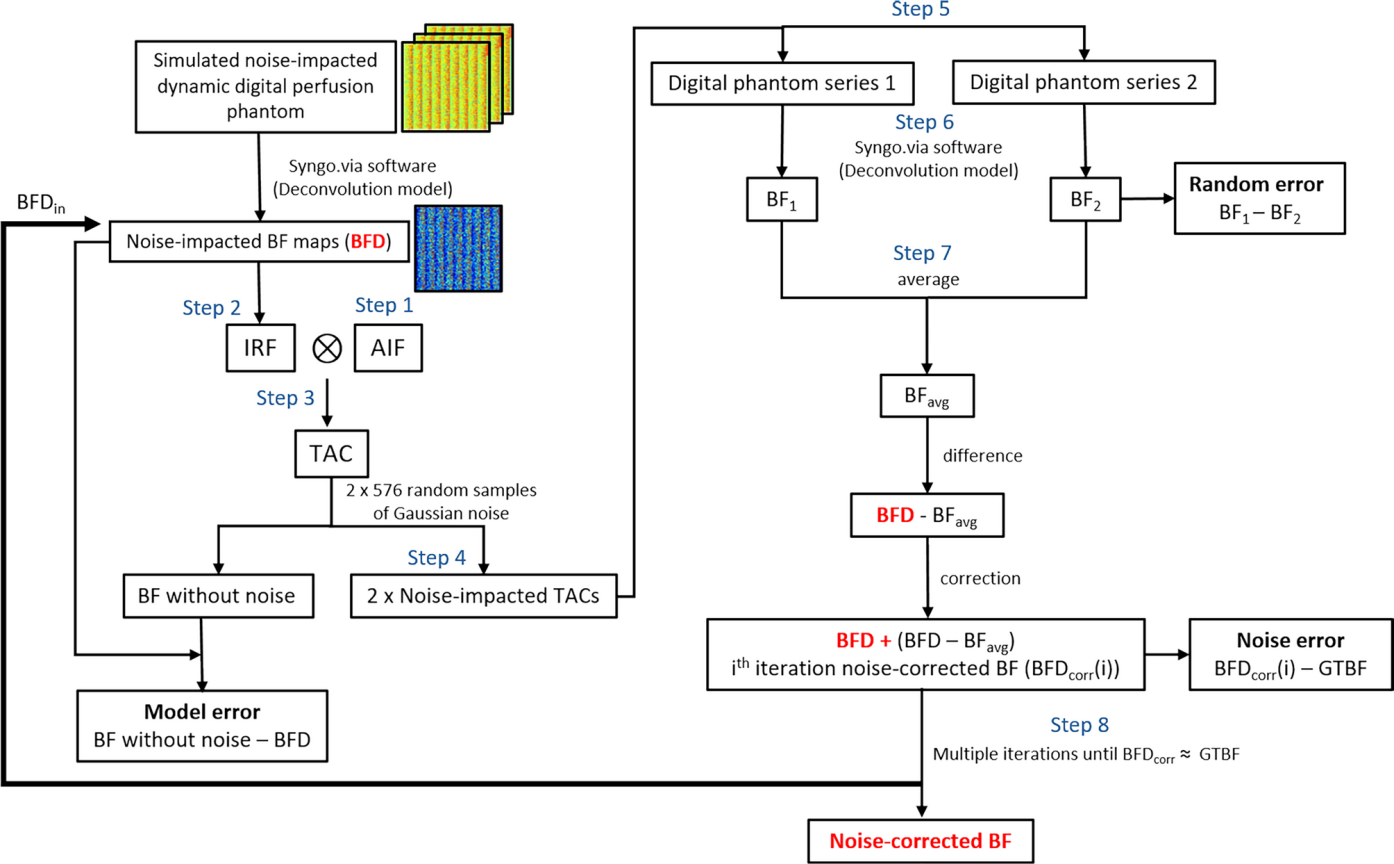
Noise error, model error, and random error curves across iterations of the noise correction algorithm.

**Fig. 3:**
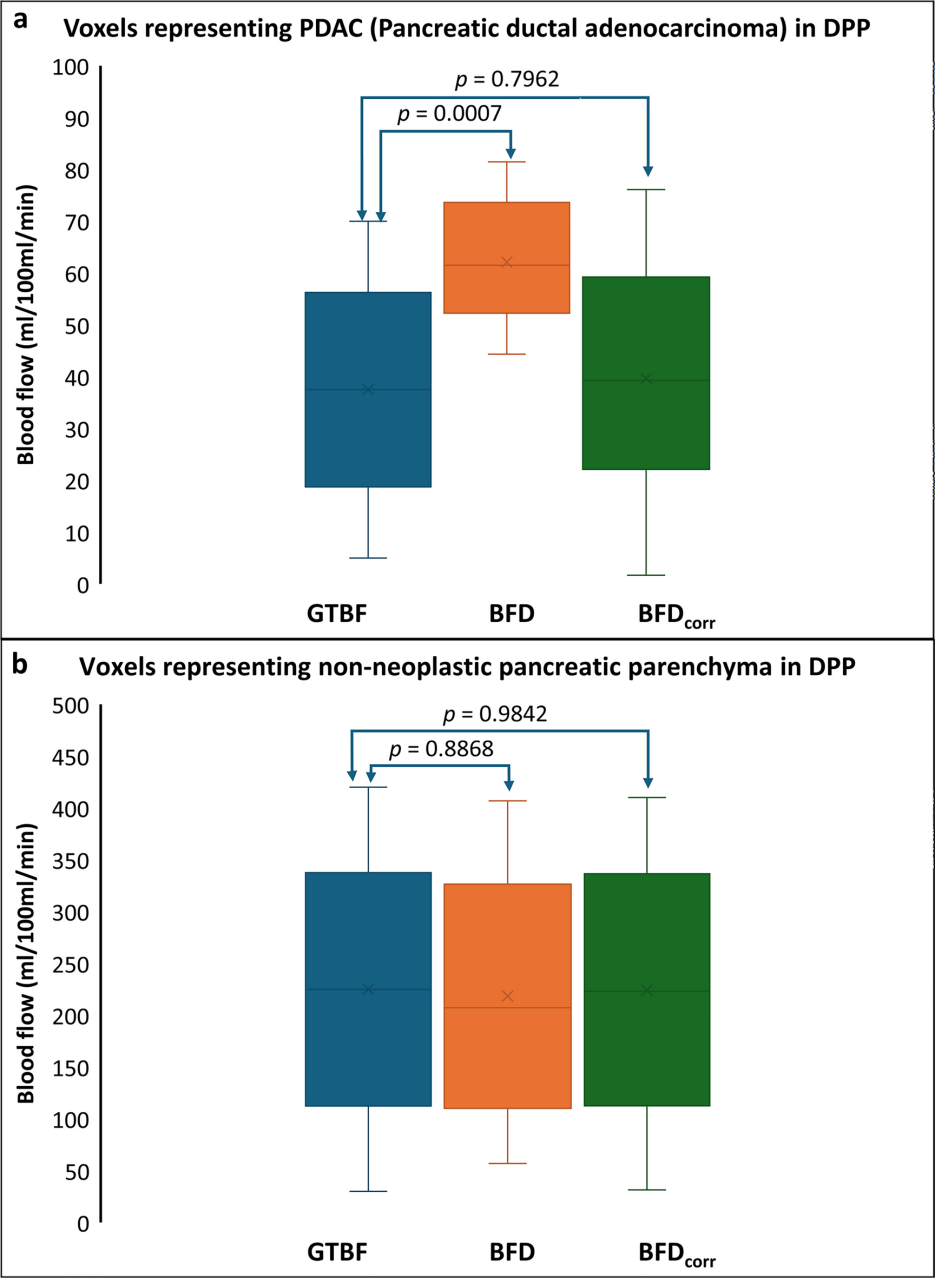
Bland–Altman plots for digital perfusion phantom (DPP) including the limits of agreement, showing a comparison between ground-truth (GTBF) and noise-corrected blood flow (BFD_corr_) measurements.

**Fig. 4:**
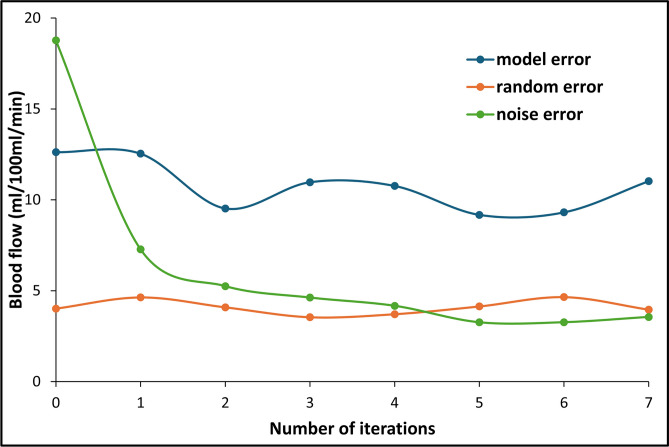
Bland–Altman plot including the limits of agreement, showing a comparison between the noise-impacted (BFD) and noise-corrected blood flow (BFD_corr_) measurements in the (**a**) Digital Perfusion Phantom and (**b**) the clinical dataset.

**Fig. 5:**
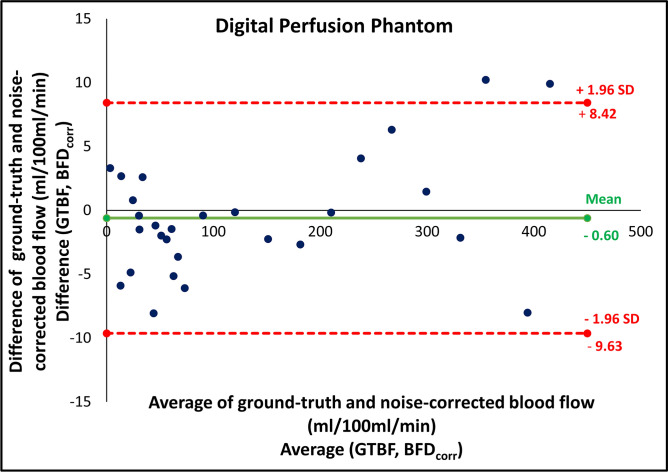
A detailed explanation of the output obtained at each step during development of the noise correction algorithm. (**a**) An arterial input function (AIF) of a digital perfusion phantom (DPP) with zero baseline and temporal sampling rate of 1.5 s, generated by averaging the AIFs from a cohort of 59 pancreatic ductal adenocarcinoma (PDAC) patients. (**b**) An example of the simulated impulse response function (IRF) of a DPP. (**c**) An example of noise-free and noise-added tissue attenuation curves (TACs) for a DPP generated for both tissue types, PDAC and non-neoplastic pancreatic parenchyma. (**d**) An example of TACs from non-neoplastic pancreatic parenchyma and PDAC regions of a PDAC patient for comparison with the simulated TACs. (**e**) An example of an image from the DICOM series of a DPP made up of 16,128 (28 sets of ground-truth values * 576 random noise samples) TACs. The top right corner of the phantom image represents the AIF. (**f**) An example of a blood flow (BF) map obtained from a DPP using commercially available CT perfusion software (syngo.via Body perfusion VB10B, Siemens Healthineers).

**Fig. 6:**
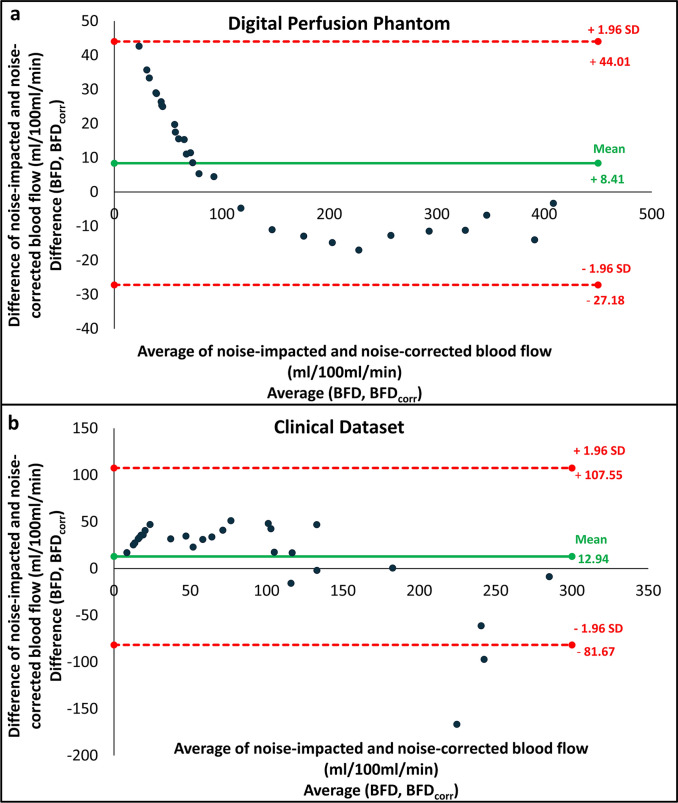
A flow chart illustrating the complete noise correction process for blood flow (BF) measurements, including evaluation using digital perfusion phantom (DPP) (starting from the first block) and evaluation using a clinical dataset (starting from the second block). For the DPP analysis, each GTBF value is simulated using two independent sets of 576 noise-impacted TACs, resulting in BF1 and BF2 estimates for random error calculation. This process is repeated for 28 GTBF values, totaling 16,128 TACs. For the clinical dataset, patient BF values calculated using the deconvolution model from Mayer’s study^18^ were used as input for the noise-impacted BF maps. BFD represents the noise-impacted BF measurements, which need to be corrected. IRF is the impulse response function, AIF is the arterial input function, TAC represents the tissue attenuation curve, and GTBF is the ground-truth blood flow. BFD_corr_(i) represents the noise-corrected BF measurement for the i^th^ iteration. The random error and model error calculations are also shown in the flow chart. This iterative process for DPP continues until BFD_corr_ aligns with GTBF or until the error between GTBF and corrected measurements is minimized to an acceptable threshold.

The original Article has been corrected.

