# Supplementary figures and images for: Model based noise correction enhances the accuracy of pancreatic CT perfusion blood flow measurements
Source: Sci Rep. 2025 Oct 23;15:37165. doi: 10.1038/s41598-025-24482-x (PMC12550029; doi:10.1038/s41598-025-24482-x)

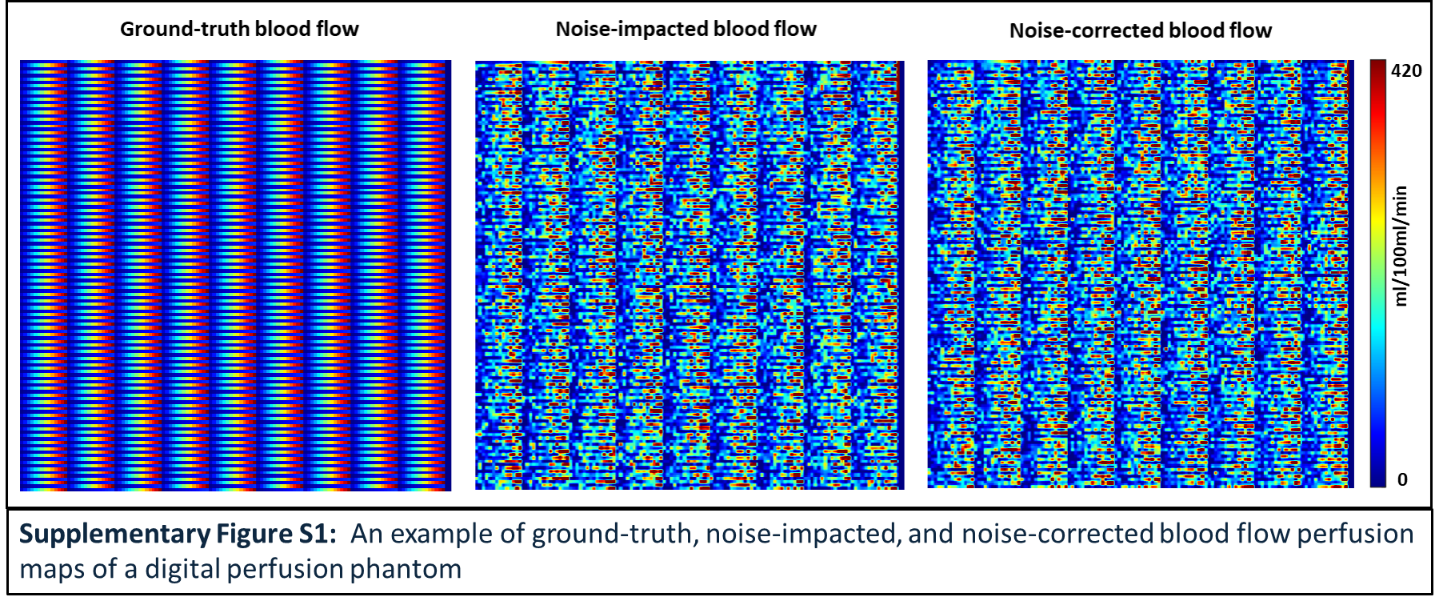

Supplement: Supplementary file 1 — Supplementary Material 1 [file 41598_2025_24482_MOESM1_ESM.docx]
